# Arthroscopic findings and treatment of maisonneuve fracture complex

**DOI:** 10.1186/s12891-021-04713-8

**Published:** 2021-09-24

**Authors:** Lukas Fraissler, Georg Mattiassich, Lars Brunnader, Lukas A. Holzer

**Affiliations:** 1 Department of Orthopedic Surgery, Klinik Diakonissen Schladming, Salzburger Strasse 777, 8970 Schladming, Austria; 2 Paracelsus Medical University Salzburg, Strubergasse 21, 5020 Salzburg, Austria; 3 AUVA Trauma Center Klagenfurt am Wörthersee, Waidmannsdorfer Strasse 35, 9020 Klagenfurt am Wörthersee, Austria

**Keywords:** Maisonneuve fracture, Tibio-fibular instability, Syndesmotic rupture, Ankle arthroscopy, Osteochondral lesion, Cartilage lesion

## Abstract

**Background:**

The Maisonneuve fracture complex (MFC) is a well-known lower leg injury. However, the optimal treatment is still not clear and there is limited data on concomitant injuries of cartilage. Therefore, the aim of our study was to report the incidence of incidental cartilage injuries and their management in arthroscopic treatment of MFC.

**Patients and methods:**

Between February 2018 and February 2021 all patients presenting with MFC in our department were treated with diagnostic ankle arthroscopy and percutaneous syndesmotic screw or suture-endobutton fixation. In case of instable cartilage, it was debrided and according to the International Consensus Meeting on Cartilage Repair of the Ankle, in grade IV lesions < 10 mm or < 100 mm^2^ area the subchondral bone was microfractured.

**Results:**

Eighteen patients, 16 male and two female, with a mean age of 48.1 years, were included. In all cases, instability of the distal tibiofibular articulation was confirmed arthroscopically. Injuries of the cartilage were found in 56% of the cases and in 31% of the patients surgical intervention was required. In three talar and one tibial lesion additional arthroscopic bone marrow stimulation with microfracture of the subchondral bone was performed.

**Conclusions:**

Ankle arthroscopy is a helpful method to guide fibular reduction and to detect and address associated cartilage injuries. Due to the high rate of chondral lesions, addressing these arthroscopically may contribute to better postoperative results.

**Level of evidence:**

IV

## Introduction

*“La fracture du péroné”,* or better known as Maisonneuve fracture complex (MFC) was first described by the French surgeon Jacques Gilles Maisonneuve in 1840 [1]. The injury typically consists of a fracture of the proximal fibula with disruption of the distal tibiofibular syndesmosis and a deltoid rupture or medial malleolus fracture.

The mechanism of the injury was described by Pankovich as a strong external rotation force with the foot in slight supination and in neutral or slight pronation in later stages [2].

Regarding therapy, some authors reported of nonoperative treatment: Pankovich [2] treated the MFC nonoperatively in cases without rupture of the deltoid ligament, interosseous ligaments, or medial malleolus fracture and Merrill [3] suggested that these are often more stable than generally assumed. According to the Lauge-Hansen classification, this could be possible, but sometimes it is difficult to differentiate between partially and total ruptured syndesmotic ligaments preoperatively. Therefore, MFC should be assumed as an unstable injury, and most authors recommend operative treatment [4–9]. Stufkens [9] defined in their review of literature recommendations for treatment of Maisonneuve fractures: the medial malleolus should be fixated, the torn deltoid ligament need not be directly repaired, syndesmotic instability can be treated with one or two 3- or 4-cortical screws which can be placed percutaneously, and the proximal fibular fracture does not require direct internal fixation. However, the optimal operative management is not clear and various options are under debate.

Moreover, Hintermann [10] and Loren [11] reported of an incidence of cartilage lesions, including chondral defects and osteochondral lesions, in ankle fractures of 79.2%, respectively 63%. Yoshimura [8] reported that all patients with MFC, who underwent ankle arthroscopy, had cartilaginous damage to the medial section of the talar dome. Therefore, we started to treat patients with Maisonneuve fracture arthroscopically to detect and address concomitant injuries. The aim of this study was to retrospectively evaluate the incidence of cartilage injuries in these patients.

## Materials and methods

Between February 2018 and February 2021 all 18 patients presenting with MFC, 16 men and two women, were treated consecutively with ankle arthroscopy in our department. The mean age at time of surgery was 48.1 years (range 23 to 74 years). Eleven MFCs were sports related, six occurred as a result of an ankle sprain while walking, and one patient had a motorcycle accident (Table 1).

**Table 1:** Patients characteristics, Surgical Intervention, Trauma mechanism, Findings

Age [years]	Sex	Side	Diagnosis (in addition to syndesmotic instability)	Localization of chondral lesion	Size [mm]	ICRS Grade	Surgical Intervention (in addition to ankle arthroscopy)	Trauma
57	Male	right	Chondral lesion, medial malleolus fracture, posterior tibia fracture	Talus medial	5 × 5	IV	Microfracture, 2x tricortical 3.5 mm stainless steel screws, ORIF medial malleolus	Skiing
46	Male	left	Medial malleolus fracture				Suture-endobutton, ORIF medial malleolus	Motorcycle riding
24	Female	right	Deltoid rupture				Suture-endobutton	Bicycling
27	Male	left	Chondral lesion	Tibia	10 × 4	IV	Microfracture, 2x tricortical 3.5 mm stainless steel screws	Ski mountaineering
52	Male	left	Chondral lesion, medial malleolus fracture, Chaput tubercle fracture	Talus medial	4 × 4	IV	Microfracture, percutaneous fixation medial malleolus, 2x tricortical 3.5 mm stainless steel screws	Walking
74	Male	right	Medial malleolus fracture, posterior tibia fracture				2x tricortical 3.5 mm stainless steel screws	Cross-country skiing
41	Male	left	Chondral lesion, posterior tibia fracture	Talus medial	4 × 4	I	2x tricortical 3.5 mm stainless steel screws	Ski mountaineering
49	Male	right	Deltoid rupture, posterior tibia fracture				2x tricortical 3.5 mm stainless steel screws	Martial Arts
55	Male	right	Chondral lesion, deltoid rupture posterior tibia fracture	Talus medial	8 × 6	IV	Microfracture, 2x tricortical 3.5 mm stainless steel screws,	Hiking
61	Male	left	Deltoid rupture				2x tricortical 3.5 mm stainless steel screws	Hiking
23	Male	left	Medial malleolus fracture, posterior tibia fracture				2x tricortical 3.5 mm stainless steel screws	Walking
40	Male	right	Osteochondral lesion, posterior tibia fracture	Tibia	N/A	IV	Debridement of unstable cartilage/bone, ORIF medial malleolus, 2x tricortical 3.5 mm stainless steel screws	Ski mountaineering
43	Male	left	Osteochondral lesion, medial malleolus fracture, posterior tibia fracture	Tibia, Talus medial	22x4x4, 10 × 5	IV, III	Debridement of unstable cartilage/bone, ORIF medial malleolus, 2x tricortical 3.5 mm stainless steel screws	Walking
33	Male	left	Deltoid rupture, posterior tibia fracture				2x tricortical 3.5 mm stainless steel screws	Walking
69	Male	right	Chondral lesion, deltoid rupture, posterior tibia fracture	Talus medial	10 × 5	IV	Microfracture, 2x tricortical 3.5 mm stainless steel screws,	Walking
63	Male	right					2x tricortical 3.5 mm stainless steel screws	Hiking
40	Male	right					2x tricortical 3.5 mm stainless steel screws	Hiking
68	Female	right	Chondral lesion	Talus medial	3 × 8	IV	Debridement of unstable cartilage/bone, 2x tricortical 3.5 mm stainless steel screws	Walking

N/A – Due to an osteochondral comminution fracture of the anterior border of the distal tibia defect sizing was not applicable

MFC was confirmed with radiographs of the ankle and knee. In 5 patients an additional CT-scan and in 3 patients an MRI of the ankle was performed.

Preoperatively, prophylactic antibiotics were given intravenously. All Arthroscopies were performed or attended by a national-board certified foot and ankle surgeon in spinal anaesthesia and tourniquet control. Therefore, patients were set in supine position, no ankle distractor was applied, and saline was injected to inflate the ankle joint. Routinely, a 4 × 152.5 mm/30° arthroscope with standard anteromedial and anterolateral portals was used to access the ankle.

**Fig. 1:**
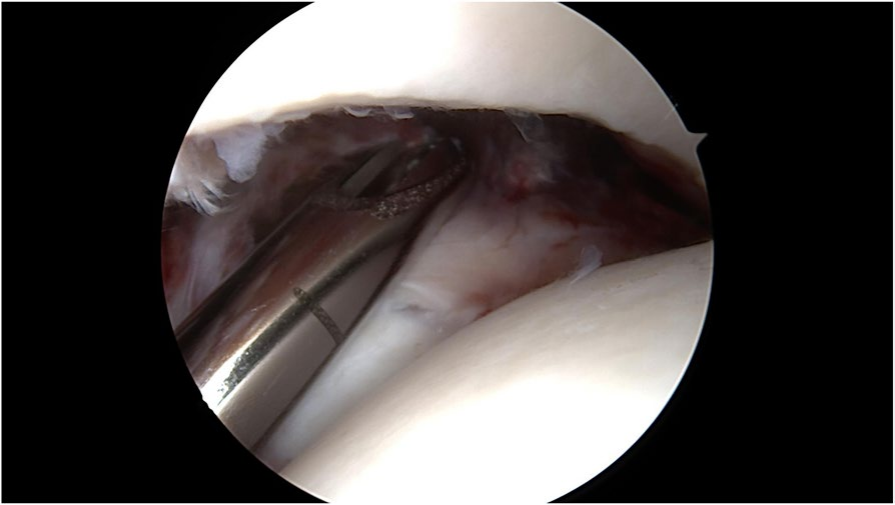
Diastasis of the distal fibula was proven with a 4 mm shaver. In all cases an apparent syndesmosis instability of at least 4 mm was seen

An anterior ankle examination, as described by Ferkel [12], was used to verify syndesmotic instability and to check for cartilage injuries. External rotation stress test was used to assess syndesmotic injury. Frank syndesmosis instability was defined by ≥2 mm displacement of the lateral malleolus (Fig. 1) [13]. Lesions of the cartilage were classified according to the International Cartilage Repair Society (ICRS) grading system [14].

Instable cartilage was debrided and, as recommended by the International Consensus Meeting on Cartilage Repair of the Ankle, in lesions < 10 mm or < 100 mm^2^ area subchondral bone was microfractured [15].

After addressing cartilage damage, avulsed ligaments and debris of the distal tibiofibular syndesmosis was resected with a 4 mm shaver to allow proper positioning of the fibula into the incisura. Closed reduction was performed with a sharp reduction clamp and controlled arthroscopically and fluoroscopically. Length to the fibula was restored and internal rotation as well as medial translation was assessed. Fixation of the fibula was either achieved with percutaneous placement of two tricortical 3.5 mm stainless steel screws or suture-endobuttons (TightRope®, Arthrex, Naples, FL).

Medial malleolar fractures were addressed with open reduction and screw fixation, whereas deltoid ligament disruptions were treated non-operatively in the context of postoperative immobilization with cast or walking boot. In cases of arthroscopy and microfracture of talus or tibia, or suture-endobutton fixation of the fibula, patients were instructed for partial weight-bearing (15 kg) for 6 weeks and with screw fixation only partial weight-bearing (15 kg) for 2 weeks, followed by 4 weeks of full weight-bearing. According to our postoperative standard protocol syndesmosis screws were removed 6–8 weeks postoperative.

## Results

In all 18 cases, a frank instability of the distal tibiofibular articulation was confirmed in intraoperative stress testing. Four patients had an arthroscopically confirmed disruption of the deltoid ligament and six patients a fracture of the medial malleolus. Furthermore, 11 patients had a radiologically approved additional posterior fracture of the distal tibia or tibial avulsion fracture of the posterior inferior tibiofibular ligament.

**Fig. 2:**
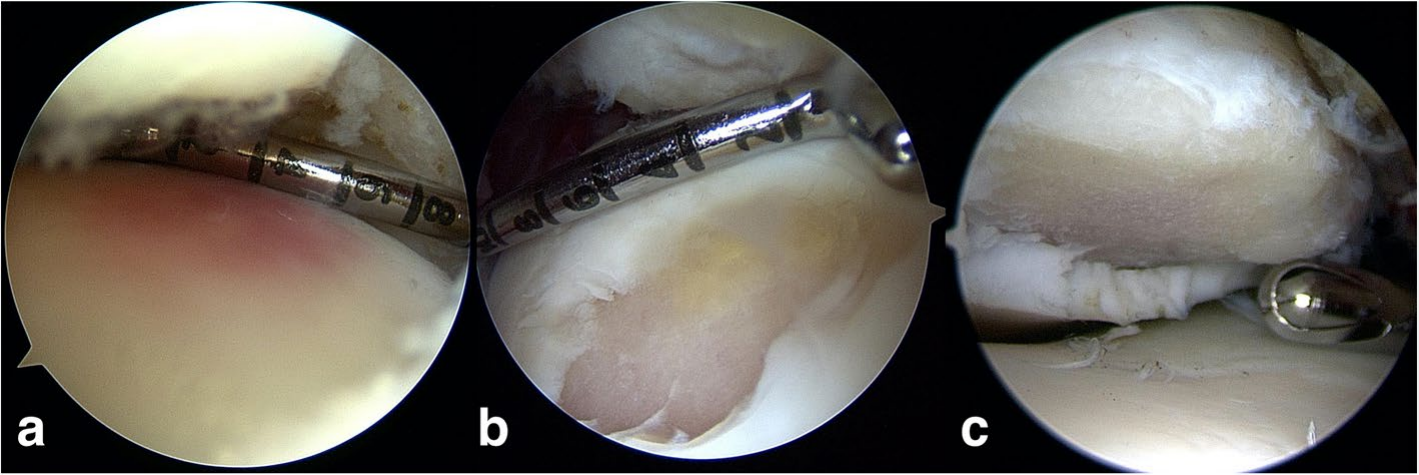
A variety of (osteo-)chondral lesions were found arthroscopically: cartilage hematoma (**a**) as well as instable talar (**b**) and tibial (**c**) cartilage injuries, which were debrided and subchondral bone microfractured

**Fig. 3:**
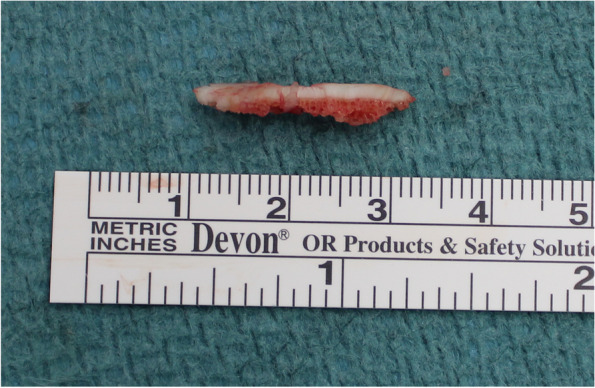
In one patient a 22x4x4 mm osteochondral fragment of the anteromedial edge of the distal tibia had to be removed

Traumatic lesions of the cartilage were found in 10 of 18 ankles (56%). The medial aspect of the talar dome was involved in eight cases, while tibial articulation was affected in 2 patients. A wide range of different (osteo-)chondral lesions was seen intraoperatively (Figs. 2 and 3) and classified as shown in Table 1. With exception of the patient with the grade I talar lesion, additional operative treatment was needed in nine cartilage lesions. Therefore, all nine patients were treated with arthroscopical debridement of ruptured and loose cartilage. In three talar and one tibial lesion additional bone marrow stimulation with microfracture of the subchondral bone was necessary.

Postoperative treatment was performed according to our standard protocol with partial weight-bearing for 6 weeks or partial weight-bearing for 2 weeks, followed by 4 weeks of full weight-bearing and screw removal after 6 weeks.

Twelve patients were available for a short-term follow-up. Mean time to follow-up was 15 months (range 3 to 27 months). No perioperative infections or wound complications occurred. After 10 months one patient needed revision surgery due to secondary syndesmosis and deltoid ligament diastasis and in one case of TightRope® fixation implant removal and arthroscopic scar debridement was necessary. None of the patients complained about secondary cartilage issues. At the time of last follow-up all patients were very satisfied or satisfied with the results.

## Discussion

Treatment of MFC varies widely; traditional therapy consists of open or percutaneous techniques with restoration of the ankle mortise and syndesmosis screw fixation. Kalyani [16] reported in a review, including 61 patients in 4 studies, of excellent results in 47.54%, good in 40.98%, fair in 4.92% and poor in 6.55% as well as an ankle arthrosis rate of 16% after an ﻿follow-up period from 25.3 months to 6.4 years. Furthermore, a high rate of associated cartilage lesions in common ankle fractures of up to 79.2% [10, 11] and 100% in MFC [8] have been described. According to this data, a high rate of concomitant cartilage injuries is confirmed in our study. Therefore, ankle arthroscopy might be a favourable method to detect and subsequently treat associated injuries.

In general, the incidence of complications in ankle arthroscopy is low. Imade [17] reported of one patient with compartment syndrome following ankle arthroscopy after MFC. In our own patient collective, as well as in the report of Yoshimura [8], were no complications as a result of ankle arthroscopy found.

Furthermore, the mechanism of injury was supposed to be a strong external rotation force with the foot in slight supination and in neutral or slight pronation in later stages [2]. Based on the fact that there was no damage to the posterior malleolus in their patient collective, Yoshimura [8] concluded that there is a strong possibility that the MFC could be a pronation external rotation type fracture according to the Lauge-Hansen classification. In four of our patients MFC occurred in a ski boot while skiing; Fritschy [18] also reported of Maisonneuve injuries in professional skiers with rigid ski boots, in which pronation or supination is theoretically impossible. Therefore, the mechanism of injury and potentially associated intra-articular damage have to be thought about.

Regarding limitations of this study, the small and heterogenous patient collective has to be disclosed. Furthermore, there was no control group, a short follow-up time and no long-time results available. On the other hand, the strength of this paper represents the largest number of study participants with MFC who were treated arthroscopically in current literature and all patients were treated by a national-board certified foot and ankle surgeon.

## Conclusions

Ankle arthroscopy is helpful to detect and treat cartilage lesions of talus and tibia in MFC, as well as to guide fibular reduction. To avoid secondary complications, we do not recommend using endobutton fixation for MFC and early screw removal. Furthermore, the long-term results of this technique compared to conventional open surgery need to be evaluated in a larger patient cohort. Nonetheless, due to the high rate of chondral lesions, addressing these arthroscopically may contribute to better postoperative results.

## Data Availability

The datasets used and/or analysed during the current study are available from the corresponding author on reasonable request.

## Abbreviations

MFC: Maisonneuve fracture complex
ICRS: International Cartilage Repair Society
